# An assessment of geographical access and factors influencing travel time to emergency obstetric care in the urban state of Lagos, Nigeria

**DOI:** 10.1093/heapol/czab099

**Published:** 2021-08-23

**Authors:** Aduragbemi Banke-Thomas, Kerry L M Wong, Lindsey Collins, Abimbola Olaniran, Mobolanle Balogun, Ololade Wright, Opeyemi Babajide, Babatunde Ajayi, Bosede Bukola Afolabi, Akin Abayomi, Lenka Benova

**Affiliations:** LSE Health, London School of Economics and Political Science, Houghton Street, London WC2A 2AE, UK; Centre for Reproductive Health Research and Innovation, Lagos State University College of Medicine, Oba Akinjobi Street, Ikeja, P.M.B. 21266, Lagos, Nigeria; Department of Infectious Disease Epidemiology, London School of Hygiene and Tropical Medicine, Keppel Street, London WC1E 7HT, UK; School of Geographical Sciences and Urban Planning, Arizona State University, South Myrtle Avenue, Tempe, Arizona 85281, USA; Department of Disease Control, London School of Hygiene and Tropical Medicine, Keppel Street, London WC1E 7HT, UK; Department of Community Health and Primary Care, College of Medicine, University of Lagos, Idi Araba, PMB 12003, Lagos, Nigeria; Centre for Reproductive Health Research and Innovation, Lagos State University College of Medicine, Oba Akinjobi Street, Ikeja, P.M.B. 21266, Lagos, Nigeria; Department of Community Health and Primary Health Care, Lagos State University College of Medicine, Oba Akinjobi Street, Ikeja, P.M.B. 21266, Lagos, Nigeria; Department of Epidemiology and Medical Statistics, University of Ibadan, Oduduwa Road, 200132, Ibadan, Nigeria; Centre for Reproductive Health Research and Innovation, Lagos State University College of Medicine, Oba Akinjobi Street, Ikeja, P.M.B. 21266, Lagos, Nigeria; Office of the Commissioner, Lagos State Ministry of Health, Secretariat, Alausa, Lagos, Nigeria; Department of Obstetrics and Gynaecology, College of Medicine of the University of Lagos, Idi Araba, P.M.B 12003, Lagos, Nigeria; Office of the Commissioner, Lagos State Ministry of Health, Secretariat, Alausa, Lagos, Nigeria; Department of Public Health, Institute of Tropical Medicine, Kronenburgstraat 43, 2000 Antwerpen, Belgium

**Keywords:** Maternal health, emergency obstetric care, universal health coverage, care-seeking, referral, geographical coverage, accessibility, travel, urban, Lagos, Nigeria

## Abstract

Previous efforts to estimate the travel time to comprehensive emergency obstetric care (CEmOC) in low- and middle-income countries (LMICs) have either been based on spatial models or self-reported travel time, both with known inaccuracies. The study objectives were to estimate more realistic travel times for pregnant women in emergency situations using Google Maps, determine system-level factors that influence travel time and use these estimates to assess CEmOC geographical accessibility and coverage in Lagos state, Nigeria. Data on demographics, obstetric history and travel to CEmOC facilities of pregnant women with an obstetric emergency, who presented between 1st November 2018 and 31st December 2019 at a public CEmOC facility were collected from hospital records. Estimated travel times were individually extracted from Google Maps for the period of the day of travel. Bivariate and multivariate analyses were used to test associations between travel and health system-related factors with reaching the facility >60 minutes. Mean travel times were compared and geographical coverage mapped to identify ‘hotspots’ of predominantly >60 minutes travel to facilities. For the 4005 pregnant women with traceable journeys, travel time ranges were 2–240 minutes (without referral) and 7–320 minutes (with referral). Total travel time was within the 60 and 120 minute benchmark for 80 and 96% of women, respectively. The period of the day of travel and having been referred were significantly associated with travelling >60 minutes. Many pregnant women living in the central cities and remote towns typically travelled to CEmOC facilities around them. We identified four hotspots from which pregnant women travelled >60 minutes to facilities. Mean travel time and distance to reach tertiary referral hospitals were significantly higher than the secondary facilities. Our findings suggest that actions taken to address gaps need to be contextualized. Our approach provides a useful guide for stakeholders seeking to comprehensively explore geographical inequities in CEmOC access within urban/peri-urban LMIC settings.

**Key messages**

- Total travel time ranged from 2–240 minutes for women who travelled directly to a public CEmOC facility and 7–320 minutes for women who sought care there as a result of a referral.
- Pregnant women who travelled to a facility in the afternoon, morning and evening were about three, two and two times more likely to travel >60 minutes to reach a public CEmOC facility that provided care to them compared to those who travelled at night.
- Those who were referred were three times more likely to travel longer than 60 minutes compared to those who went directly to the destination facility.
- Using a Global Positioning System navigation software provided closer-to-reality travel time estimates, which when aggregated provided highly relevant insights that identify specific areas of inequity.

## Background

Maternal mortality remains a huge challenge for many health systems globally, despite a 38% reduction in global maternal mortality between 2000 and 2017. According to the World Health Organization (WHO), ∼830 women die from preventable causes related to pregnancy and childbirth every day, totalling 295 000 deaths annually. The burden is significantly higher in low- and middle-income countries (LMICs), where 99% of these maternal deaths occur. Nigeria, with an estimated 67 000 annual maternal deaths, accounts for 23% of the total global burden of maternal deaths, ranking second only to India on the list of countries with the highest number of maternal deaths (WHO, UNICEF, UNFPA *et al*., 2019). Most of these deaths occur due to five complications of pregnancy and childbirth: hypertensive disorders, obstructed labour, severe bleeding, severe infection and complications of abortion. Provision of emergency obstetric care (EmOC), which consist of nine clinical and surgical evidence-based interventions, is effective in managing these complications (Paxton *et al.*, 2005). Seven of these interventions (parenteral antibiotics, uterotonic drugs, parenteral anticonvulsants, manual removal of placenta, removal of retained products of conception, assisted vaginal delivery and neonatal resuscitation) are classified as basic emergency obstetric care (BEmOC). In addition to BEmOC interventions, blood transfusion and surgery (i.e. caesarean section and exploratory laparotomy) complete the comprehensive emergency obstetric care (CEmOC) package (WHO, UNFPA, UNICEF *et al.,* 2009). Prompt access to EmOC provided by skilled health personnel reduces maternal deaths amongst women who reach health facilities by 15–50% and intrapartum stillbirths by 45–75% (Paxton *et al.*, 2005; WHO, UNFPA, UNICEF *et al.*, 2018).

However, before pregnant women with obstetric emergencies can access these interventions, they need first to decide that it is time to seek care (Phase I) and then travel to appropriate facilities (Phase II). They then need to be promptly managed when they arrive at these facilities (Phase III). During these phases, pregnant women can experience delays that further increase the risk of poor pregnancy outcomes for them and their babies (Thaddeus and Maine, 1994). Of particular interest in this paper is delays that occur during Phase II. For this, pregnant women in many LMICs often have to make it to the health facility by themselves or with the help of their relatives (Afari *et al.*, 2014; Banke-Thomas *et al.*, 2020). What has been well established is that travel time from home to a health facility has a significant impact on pregnancy outcomes for mothers and newborns (Ravelli *et al.*, 2011; Wilson *et al.*, 2013). In some LMIC settings, including Lagos, the highest proportion of maternal deaths occurred due to delays during Phase II (Okonofua *et al.*, 2017; Chavane *et al.*, 2018).

In 2009, the WHO stated that EmOC facilities should be ‘available within 2–3 hours of travel for most women’ was a reasonable target for health systems. They suggested that analyses to estimate this travel time should be conducted as part of a supplementary study assessing the EmOC indicator focused on geographical accessibility and distribution of facilities (WHO, UNFPA, UNICEF *et al*., 2009). The 2015 Lancet Commission on global surgery defined geographical accessibility as the proportion of the population that can access a health facility with the capacity to provide essential surgical and anaesthesia services, including caesarean section, within 2 hours. The commission set a target of 80% as the minimum coverage to be achieved by 2030 (Meara *et al.*, 2015). However, complications of pregnancy and childbirth can result in rapid deterioration, ‘in less than 2 hours’, and for some women, even in minutes (Khan and El-Rafaey, 2006; UNFPA, 2012).

Several studies that have assessed travel time of pregnant women in LMICs to reach EmOC facilities have mostly been based on self-reported estimates from the women themselves (small scale) or spatial models using geographic information systems (GIS) analysis (often large scale) (Gething *et al.*, 2012; Chowdhury *et al.*, 2017; Banke-Thomas *et al.*, 2019; 2020; Makacha *et al.*, 2020; Mubiri *et al.*, 2020; Rudolfson *et al.*, 2020). However, the accuracy of these approaches in reflecting actual travel time has been questioned. On the one hand, self-reported travel time using women’s report carries substantial limitations to accuracy. These include recall bias (difficulty to accurately recollect time in the case of an emergency), survival bias (women who died do not report) and heaping as issues compromising the validity of estimates based on self-reported travel time for widespread use as a data source for these important accessibility indicators (Rudolfson *et al.*, 2020). On the other hand, GIS model-based studies typically estimate travel time based on the certain nominal overall speed of movement (Weiss *et al.*, 2018). These GIS model-based approaches are readily extendible and scalable. They have been used to estimate the time required for the whole population or certain target groups (e.g. women and older people) to reach emergency care, surgical care and EmOC, among others (Gething *et al.*, 2012; Chowdhury *et al.*, 2017; Banke-Thomas *et al.*, 2020; Makacha *et al.*, 2020; Mubiri *et al.*, 2020). The shortest path algorithm is applied to obtain travel time estimates between the target population and the nearest point of care. In the case of pregnant women in LMICs, however, women often bypass their nearest facility for various reasons, leading to under-estimation (Kruk *et al.*, 2009; Salazar *et al.*, 2016; Shah, 2016; Keyes *et al.*, 2019; Banke-Thomas *et al.*, 2020; Makacha *et al.*, 2020; Mubiri *et al.*, 2020). In addition, these estimation methods currently offer little flexibility to account for important travel conditions that can severely impede travel, such as time of day, day of the week, delays due to traffic, poor or inaccessible road infrastructure, roadworks and security issues such as roadblocks. These limitations minimize the usefulness of such assessments for policymakers and service planners.

Global Positioning Satellite (GPS) navigation software applications such as Google Maps and Waze have been in existence since the mid-2000s (Boulos, 2005). Both have since been used to estimate travel time, by type of transport, as part of our everyday living (Wang and Xu, 2011). Like the modelling approaches mentioned above, Google Maps estimates of motorized travel time are based on official speed limits and likely speed derived from road type. In addition, historical average speed data over certain time periods, actual travel times crowdsourced from previous users, live traffic and road closures information also come into play. For health service access research, Google Maps has been used in high-income settings to use more realistic travel time estimates to reach health facilities (Kelly *et al.*, 2016). A recent study showed that compared to estimates by GIS-modelled platforms, Google Maps allowed the best-case estimate of reality in an urban LMIC setting (Banke-Thomas *et al.*, 2021). Such closer-to-reality estimates need to be deployed to support robust planning for health service delivery at scale. The objectives of this study were to (1) estimate more accurate travel times for pregnant women who presented with an obstetric emergency at public CEmOC facilities using Google Maps, (2) assess system-level factors influencing travel time and (3) use the improved travel time estimates to assess geographical accessibility and coverage of CEmOC in Lagos state, Nigeria.

## Materials and methods

### Study location

**Figure 1.**
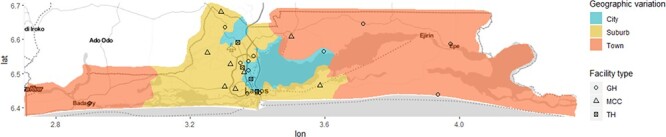
Map of Lagos state showing variation in geographical settlements and location of the public CEmOC facilities.

The study was conducted in Lagos State, southwest Nigeria. The Lagos State Bureau of Statistics estimated that 25.6 million people resided in Lagos State in 2019: a density of 6871 residents per square kilometre (km) (LASG, 2019). The state is further divided into 20 local government areas (LGA). Lagos state is highly urbanized and has a mix of different geographical terrains, including city and suburb, metropolis and slums, as well as land and riverine areas. The central areas form the Lagos metropolis, which is surrounded by several suburbs. In contrast, the extreme western and eastern parts of the state are made up of less built-up towns (Figure 1).

Compared to the national maternal mortality ratio (MMR) of 512 maternal deaths per 100 000 live births (year—2017) (National Population Commission, ICF International, 2019), MMR in Lagos State has been estimated as 450 (95% CI 360–530) per 100 000 live births (Oye-Adeniran *et al.*, 2011). When disaggregated by LGAs, MMR ranges from 356 per 100 000 live births in Ikeja LGA to 826 per 100 000 live births in Alimosho LGA. Similar to global patterns, hypertension, spontaneous abortions and ectopic pregnancies were the most commonly reported causes of death during pregnancy, while haemorrhage and prolonged or obstructed labour were more commonly reported as causes of death during childbirth in Lagos (Odeyemi *et al.*, 2014; Okonofua *et al.*, 2017).

In terms of available CEmOC facilities, there are 24 public CEmOC facilities in Lagos State, including 20 secondary health care facilities (general hospitals/maternal childcare centres) and four tertiary health care facilities (teaching hospitals/apex referral centres), all of which are expected to provide CEmOC services 24 hours a day (Figure 1 and Supplementary Data). There is also a complement of three military hospitals and about 35 private hospitals that can be classified as CEmOC facilities with specialists who can provide CEmOC services 24 hours a day, as per the database of the State Ministry of Health. However, for this study, we focus only on public sector hospitals providing CEmOC, as they form the bedrock of universal health coverage in LMICs (Sachs, 2012). In any case, as per 2018 Nigeria Demographic Health Survey (NDHS), excluding home delivery (59.0%), twice the number of women in Nigeria deliver in public hospitals (26.4%) compared to private hospitals (13.0%) (National Population Commission, ICF International, 2019).

### Data collection

Data for this study were collected over 6 months and based on a review of patient records of all pregnant women who presented in the obstetric emergency rooms of all 24 public CEmOC facilities in Lagos state with any major pregnancy and childbirth complication between 1st November 2018 and 30th October 2019. However, some facilities were being built or renovated during this one-year study period. First, the Institute of Maternal and Child Health (IMCH, commonly referred to as Àyìnkę House) was only re-opened for service after a 9-year closure for renovation on 24th April 2019 (Okoghenun, 2016; Ugvodaga, 2019). As such, we could only extract 3 months of data from the facility (1st July 2019 to 30th September 2019). Second, Eti-Osa Maternal and Child Care Centre (MCC) was newly built and commissioned (Bassey, 2019). Data collected from this facility were for the period 1st September 2019 to 31st December 2019. The data were mined from the records by members of the research team supported by trained research assistants who were qualified medical doctors conversant with the patient records system in Lagos public health facilities.

Using a pre-tested data extraction tool, we collected data on demographic characteristics, obstetric history, travel to reach the health facility (month of the year, day of the week—weekday or weekend and period of day when the journey to the facility commenced—morning, afternoon, evening or night), street name of women’s self-reported start location (place of residence, unless otherwise stated), other facilities visited en route (referral points) if any and the destination facility (one of the 24) from clerking notes recorded in the patient folders. In cases where clarification was needed, we solicited the support of the medical doctors in charge of the obstetric emergency room.

We geo-coded the origin, any facilities visited along the way (referral points) and destination locations for each woman. The points of origin were based on the stated street name of the women’s self-reported start location, most commonly her home. We used Google Maps to find the exact location and selected the relevant coordinates if the street was discoverable on the platform. For streets that were difficult to find, we used local persons who were familiar with the various communities to check for any spelling errors and re-attempted to locate the street. If, despite our best efforts, we could not locate the street, the record of the woman, along with those that did not have an address marked in their patient files, was labelled untraceable (4% of the sample).

We identified the exact entry point of the obstetric emergency ward for each destination CEmOC facility by visiting the obstetric emergency ward of each facility and geocoding its location. Geographical coordinates of the CEmOC facilities were collected using a free mobile application, ‘Easy GPS’ (TopoGrafix, Stow, Massachusetts, USA), which automatically logged longitude and latitude values of the CEmOC facilities (see Supplementary Data). In cases in which pregnant women went to a referral point on her path to a CEmOC facility, we used the same approach as was used for geocoding the points of origin to geocode such referral points. However, for those who had multiple referral points, we only traced their journeys from their places of residence to the facility from where they were referred to the final destination. Stopovers made to informal settings (e.g. church or mosque) were not geo-coded.

For pregnant women whose journeys could be traced, estimated travel time between the origin and destination (including referral points) were extracted from Google Maps using the ‘typical time of travel’ tool for the time and day that the woman commenced her journey to the CEmOC facility. Motorized vehicle was used as means of transport in Google Maps, as private cars (25%) followed by taxis (21%) are the most popular means of transportation to health facilities in Lagos, as per the 2018 NDHS (National Population Commission, ICF International, 2019). While journeys that required travel by boat were identified (0.14% of the sample), these could not be traced on Google Maps. To collect travel time estimates for the period of the day when journey to the facility commenced, we used 9.00 a.m., 3.00 p.m., 6.00 p.m. and 9.00 p.m. time slots for morning, afternoon, evening or night journeys, respectively. For journeys in which we could not tell the time of the day that women commenced their journeys to the facility (33% of the sample), travel time was extracted for the afternoon (3.00 p.m.), as it offered a middle-ground estimate in between the two known peak periods for travel in Lagos (6.30 a.m. and 11.30 am—morning peak period and 3.00 p.m. and 7.30 pm—evening peak period) (Asiyanbola *et al.*, 2012).

Most up to date (as of 2017) shapefiles capturing administrative boundaries, population, road networks, and water bodies within Lagos were retrieved from the State’s Ministry of Urban and Regional Planning. These files formed the platform for which the geographical analysis was conducted.

### Data analysis

Categorical variables, which included demographic data, obstetric and travel history of the included women, were summarized using frequencies and proportions and presented in summary tables. Continuous variables were summarized using means and medians with their interquartile ranges (IQR).

Individual-level, pregnancy-related and health systems-related factors as groups of independent factors that can be associated with travel time (Sacks *et al.*, 2016; Geleto *et al.*, 2018; Ahmed *et al.*, 2019; Banke-Thomas *et al.*, 2019). Individual-level and pregnancy-related factors relate to socio-demographic and obstetric history, respectively, while health system factors comprise referral, skilled health personnel and type of facilities providing care. In addition, the season, day, period of the day when the journey took places and road conditions may also impact women’s total travel time to reach care. As our study objective was focused on system-level factors, we did not report individual-level and pregnancy-related factors as part of our analysis. We used Chi-square test (bivariate analysis) to test the null hypothesis that there is no association between day, period of day or health systems factors with reaching (or not reaching) the destination CEmOC facility within the 60 minutes. The choice of 60 minutes as benchmark, as opposed to 120 minutes, was based on the established evidence that pregnant women with obstetric emergencies can escalate in less than 2 hours (Khan and El-Rafaey, 2006; UNFPA, 2012) and that further delays could occur upon reaching the CEmOC facilities (Thaddeus and Maine, 1994; Gabrysch and Campbell, 2009). As such, a narrower window of travel will be helpful for effective service planning and policymaking decisions. In any case, other authors have used the 60 minute travel time benchmark for analysis (Chowdhury *et al.*, 2017; Niyitegeka *et al.*, 2017; Ouma *et al.*, 2018). For our analysis, associations between the independent and dependent variables were tested at a 95% confidence interval (CI), with a *P*-value of significance set at ≤0.05.

Multivariate analysis was conducted to identify the factors associated with the travel time category to reach health facilities. Using the actual travel time and the distance to reach each facility, a linear regression model was conducted to show any statistically significant differences in mean travel time and distance to CEmOC facilities. Linear regression was also conducted to compare mean travel time and distance to CEmOC facilities for women living in the area surrounding the newly established facility before and after its commissioning.

In cases where specific data were not retrieved from the patient records, such missing data were excluded from the analysis. All statistical analyses were done using STATA SE 15.0® (StataCorp, College Station, Texas, USA).

For visualization, we visually identified locations of high concentration of long travel time of >60 or >120 minutes to the destination facility. Data points were disaggregated by day of the week, period of the day, and referral. All maps were drawn with the ‘gg’ package, including the tile server for Stamen Maps, in R version 4.0.2 (R Development Core Team, Auckland, New Zealand). Data layers were projected into the spatial reference frame, WGS84/ UTM Zone 35S.

### Ethical considerations

Ethical approval for this study was obtained from the Research and Ethics Committees of the Lagos State University Teaching Hospital (LASUTH) (LREC/06/10/1226) and Lagos University Teaching Hospital (LUTH) (ADM/DCST/HREC/APP/2880). Social approval for the study was received from the Lagos State Government (LSHSC/2222/VOLII/107). As this study was based on patient records, we minimized the risk of patient identification by not collecting data on patient names and specific street numbers. In mapping, we selected the mid-point of streets of origin to ensure anonymity. Random displacements of the sort are typically used in similar large surveys (Burgert *et al.*, 2013).

## Results

In all, records of 4181 pregnant women who presented in public CEmOC facilities in Lagos state with obstetric emergencies were included in this study. Age ranged from 14 to 57 years, with mean and median age of 30 years (IQR 26–34). Journeys (minimum of origin and destination) of 4005 (95.8%) pregnant women were traceable, ranging from 42 who arrived at the IMCH (Àyìnkę House) to 541 at Epe General Hospital. The number of women with obstetric emergencies presenting in the CEmOC facilities varied across the months. Regarding the journeys to the facilities, 3233 (77%) travelled on a weekday, 1021 (24%) commenced their journey to the facility in the morning and 3144 (75%) travelled directly to the facility of care. While the remaining 1037 (25%) were referred from at least one facility and 252 (1%) had multiple stops during their referrals. Of those referred, 425 (41%) were referred from a primary health centre (PHC). For various reasons, including the unavailability of incubator spaces or need for complex surgery, 51 (1%) were referred after EmOC had been provided to them in the destination facility (Table 1). The highest proportion of women presented with bleeding [1616 (39%)]. This was followed by abdominal pain [1329 (32%)], prolonged labour [986 (24%)], high blood pressure [867 (21%)], fatigue/general tiredness [501 (12%)], not feeling foetal movement [361 (9%)], fever [323 (8%)], abortion [234 (6%)] and convulsion [193 (5%)]. Other symptoms including loss of consciousness, headache, foetal malpresentation and blurring of vision were reported in <2% of the cases.

**Table 1.** Summary of journey to care of all pregnant women who presented in public CEmOC facilities in Lagos State while in emergency situations between November 2018 and December 2019 (*n* = 4181)

**Background characteristics**	***n***	**%**	**95% CI**
Month of presentation			
January	323	7.7	7.0–8.6
February	287	6.9	6.1–7.7
March	383	9.2	8.3–10.1
April	411	9.8	9.0–10.8
May	408	9.8	8.9–10.7
June	346	8.3	7.5–9.2
July	283	6.8	6.1–7.6
August	405	9.7	8.8–10.6
September	316	7.6	6.8–8.4
October	383	9.2	8.3–10.1
November	350	8.4	7.6–9.3
December	286	6.8	6.1–7.7
Day that journey to facility commenced			
Weekend	948	22.7	21.4–23. 9
Weekday	3233	77.3	76.0–78.6
Period of day that journey commenced			
Morning	1021	24.4	23.1–25.8
Afternoon	751	18.0	16.8–19.2
Evening	644	15.4	14.3–16.5
Night	397	9.5	8.6–10.4
Missing	1368	32.7	31.3–34.2
Means of travel to the facility			
Private car	28	0.7	0.5–2.0
Taxi	13	0.3	0.2–0.5
Bus	13	0.3	0.2–0.5
Tricycle	13	0.3	0.2–0.5
Motorcycle	3	0.1	0.0–0.2
Missing	4111	98.3	97.9–98.7
Referral			
Not referred	3166	75.7	73.9–76.5
Referred	1015	24.3	23.5–26. 1
Type of referral institution			
Another hospital (public)	164	15.8	13.8–18.3
Another hospital (private)	238	23.0	21.5–25.6
Clinic (public or private)	79	7.6	6.1–9.4
PHC	425	41.0	37.9–44.0
Traditional birth attendant	103	9.9	8.2–11.9
Non-health facility (church, mosque)	22	2.1	1.4–3.2
Nursing/maternity home	5	0.5	0.2–1.2
Multiple referrals (two or more)			
No	4129	98.8	98.4–99.1
Yes	252	1.2	0.9–1.6
Stop-over en route			
Non-health facility (church, mosque)	22	0.5	0.3–0.7
Ultrasound facility	4	0.1	0.0–0.2
Not reported/no stop-over	4155	99.4	98.2–99.8
Referred to another facility after receiving care			
No	4130	98.8	97.4–99.0
Yes	51	1.2	1.0–2.6

Among the women with traceable journeys, the distance travelled from origin to the destination facility ranged from 1 to 138 km for those who travelled directly to the facility and between 2 and 273 km for those who were referred. Total travel time was within 60 minutes for 3221 [80% (95% CI 79–82)] of the total sample, while for 3864 [96% (95% CI 96–97)] it was within 120 minutes. Total travel time ranged from 2 to 240 minutes for women who travelled directly to a CEmOC facility and 7 to 320 minutes for women who required referral. The median travel time to reach CEmOC facilities ranged from 8 minutes to Agbowa General Hospital to 100 minutes to IMCH (Àyìnkę House) for pregnant women who were referred. In contrast, median travel time for non-referred women ranged from 7 minutes to Somolu General Hospital to 60 minutes to reach Ibeju-Lekki General Hospital (Table 2).

**Table 2.** Included public CEmOC facilities, number of records and travel time to the facilities in minutes

	**Referral**				**No referral**				
**Facility of care**	***n***	**Mean**	**Median**	**IQR**	***n***	**Mean**	**Median**	**IQR**	**Total**
Agbowa General Hospital	10	29	8	6–32	49	27	12	6–45	59
Ajeromi Ifelodun General Hospital	44	52	27	14–71	122	19	14	10–18	166
Alimosho General Hospital	57	76	56	38–90	142	40	35	20–50	199
Amuwo-Odofin MCC	14	41	34	24–54	68	29	23	12–40	82
Apapa General Hospital	15	47	42	0–85	134	27	18	3–40	149
Badagry General Hospital	4	32	22	21–44	112	57	45	18–85	116
Epe General Hospital	160	37	15	9–43	381	24	10	5–20	541
Eti-Osa MCC^a^	31	33	35	20–45	168	32	27	16–40	199
FMC Ebute-Metta	37	59	54	44–63	80	34	33	14–45	117
Gbagada General Hospital	38	43	27	22–46	51	30	24	18–30	89
Harvey Road Health Centre	24	40	24	15–37	173	27	22	14–30	197
Ibeju-Lekki General Hospital	11	64	70	35–100	169	57	60	20–85	180
Ifako-Ijaiye MCC	4	57	55	35–80	62	47	40	13–55	68
Ijede Health Care Centre	9	36	35	20–40	86	38	40	14–55	95
Ikorodu MCC	107	46	35	18–75	293	22	18	8–30	400
IMCH—Àyìnkę House^a^	29	100	100	45–130	13	54	45	26–60	42
Isolo MCC	28	62	59	47–78	168	33	28	16–45	196
Lagos Island Maternity Hospital	184	78	70	35–102	264	45	40	18–65	448
Lagos University Teaching Hospital, Idi-Araba	82	74	61	34–100	42	48	43	22–65	124
Mushin General Hospital	17	19	13	8–18	171	11	9	6–14	188
Onikan Health Care Centre	18	51	28	22–70	77	30	16	9–45	95
Orile Agege General Hospital	72	46	41	27–56	112	24	22	14–31	184
Randle General Hospital (Gbaja-Surulere MCC)	7	32	31	23–39	96	12	11	8–14	103
Somolu General Hospital	8	23	21	8–37	111	12	7	5–15	119

a Data were collected from most facilities between 1st November 2018 and 30th October 2019. Due to construction/repair work, data were collected during different periods for the IMCH (1st July 2019 and 30th September 2019) and Eti-Osa MCC (1st September 2019 to 31st December 2019).

In bivariate analysis, travel-related (period of the day that journey commenced) and health systems factors (referral and type of referral institution) were found to be significantly associated with travel of over 60 minutes. In multivariate analysis, those who commenced their journeys in the afternoon were about three (95% CI 1.76–3.92) times more likely to travel longer than 60 minutes to reach a destination CEmOC facility compared to those who travelled at night. Those who commenced their journeys in the morning and evening were both twice as likely to travel longer than 60 minutes to reach a CEmOC facility that provided the care that they needed, compared to those who travelled at night. Those referred were three (95% CI 2.54–3.56) times more likely to travel longer than 60 minutes compared to those who went directly to the destination facility. Specifically, those who were referred from another public CEmOC facility were three and a half (95% CI 2.34–5.14) times more likely, while those from traditional birth attendants were about 69% (95% CI 0.21–0.73) less likely to travel more than 60 minutes compared to those referred from a PHC (Table 3).

**Table 3.** Bivariate and multivariate analyses of factors associated with travel time >60 minutes

**Background characteristics**	**Total**	**Travel time benchmark**		***P*-value**	**Odds ratio**	**95% CI**		***P*-value**
		**Within 60 minutes**	**Over 60 minutes**			**Lower**	**Higher**	
Day of presentation	*n* = 4005							
Weekend	912	750 (82.2%)	162 (17.8%)	0.61	–	–	–	–
Weekday	3093	2471 (79.9%)	622 (20.1%)		–	–	–	–
Period of day that journey commenced	*n* = 4005							
Morning	986	805 (81.6%)	181 (18.4%)	<0.001	2.048	1.395	3.069	<0.001
Afternoon	716	557 (77.8%)	159 (22.2%)		2.600	1.757	3.921	<0.001
Evening	613	502 (81.9%)	111 (18.1%)		2.014	1.338	3.082	<0.001
Night	374	337 (90.1%)	37 (9.89%)		1.000			
Could not tell	1316	1020 (77.5%)	784 (22.5%)					
Referral	*n* = 4005							
No	2978	2543 (85.4%)	435 (14.6%)	<0.001	1.000			
Yes	1027	678 (66.0%)	349 (34.0%)		3.009	2.544	3.559	<0.001
Type of referral institution	*n* = 1004							
Another hospital (public)	163	63 (38.7%)	100 (61.3%)	<0.001	3.463	2.336	5.141	<0.001
Another hospital (private)	236	165 (69.9%)	71 (30.1%)		0.939	0.653	1.345	0.72
Clinic (public or private)	78	54 (69.2%)	24 (30.8%)		0.970	0.548	1.677	0.91
PHC	420	288 (68.6%)	132 (31.4%)		1.000			
Traditional birth attendant	102	86 (84.3%)	16 (15.7%)		0.406	0.214	0.732	0.002
Nursing/maternity home	5	4 (80.0%)	1 (20.0%)		0.545	0.011	5.587	0.58

**Figure 2.**
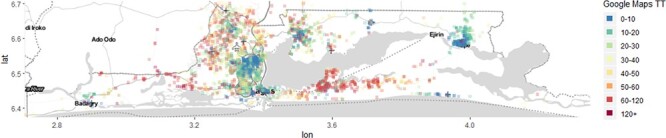
Map of Lagos state showing clusters where pregnant women needed longer than 60 minutes to travel to public CEmOC facilities.

From the mapping, it appeared that many of the pregnant women in the central cities, as well as towns to the east and west of Lagos state, tended to travel to CEmOC facilities within and around their geographical space (Figure 2). However, within the Lagos suburbs, there were three hotspots from which pregnant women needed longer than 60 minutes to travel directly to CEmOC facilities. These areas were Alimosho/Ifako-Ijaiye (Cluster A), Eti-Osa (Cluster B) and Ijanikin/Morogbo (Cluster C). These hotspots remained constant irrespective of the day of the week and period of day when the journey to the facility commenced. However, longer travel times were seen in these hotspots in the morning and afternoon. With a referral, we found that there were larger hotspots in the three suburbs from which pregnant women needed longer than 60 minutes to travel directly, i.e. Cluster A, B and C and an additional, small cluster north of Ikorodu (Cluster D) (Figure 2 and Supplementary Figures S1 and S2).

Mean travel time and distance to each CEmOC facility were compared to Agbowa General Hospital (reference point). For travel time, some facilities located in the city [Lagos Island Maternity Hospital (LIMH), IMCH (Àyìnkę House)], suburbs (Alimosho General Hospital, FMC Ebute-Metta, Ifako-Ijaiye MCC, LUTH, Mushin General Hospital, Randle General Hospital (Gbaja-Surulere MCC), Somolu General Hospital) and towns (Badagry General Hospital, Ibeju-Lekki General Hospital required statistically significant additional time of travel to access. Compared to Agbowa General Hospital, the two tertiary referral facilities [IMCH (58 (95% CI 44–72) minutes] and LUTH [38 (95% CI 27–49) minutes)] and LIMH [31 (95% CI 21–41) minutes] required the highest significant additional time to access. Also, compared to Agbowa General Hospital, distance to Ibeju-Lekki General Hospital [16.5 (95% CI 11.9–21.0) km], LIMH [8.2 (95% CI 4.1–12.4) km] and IMCH (Àyìnkę House) [7.8 (95% CI 1.7–13.9) km] was significantly longer (Table 4).

**Table 4.** Linear regression model of travel time and distance to public CEmOC facilities

**Travel time (in minutes)**					
**Facility of care^a^**	**Coef.**	**Std. Err.**	***P* > |t|**	**[95% CI]**	
Ajeromi Ifelodun General Hospital	0.3	5.4	0.96	−10.3	10.8
Alimosho General Hospital	22.6	5.3	<0.001	12.3	32.9
Amuwo-Odofin MCC	3.4	6.1	0.57	−16.9	15.3
Apapa General Hospital	1.0	5.5	0.86	−9.7	11.7
Badagry General Hospital	28.1	5.7	<0.001	17.0	39.2
Epe General Hospital	0.4	4.9	0.93	−9.1	9.9
Eti-Osa MCC	4.8	5.3	0.36	−5.5	15.1
FMC Ebute-Metta	14.2	5.7	0.01	3.1	25.3
Gbagada General Hospital	8.1	6.0	0.17	−3.6	19.8
Harvey Road Health Centre	0.7	5.3	0.89	−9.6	11.0
Ibeju-Lekki General Hospital	30.0	5.3	<0.001	19.5	40.4
Ifako-Ijaiye MCC	22.7	5.9	<0.001	11.2	34.3
Ijede Health Care Centre	10.5	5.9	0.07	−1.0	22.1
Ikorodu MCC	1.2	4.9	0.80	−8.5	10.9
Isolo MCC	9.8	5.3	0.06	−0.5	20.2
Lagos Island Maternity Hospital	31.1	4.9	<0.001	21.5	40.7
IMCH—Àyìnkę House	58.4	7.2	<0.001	44.4	72.5
Lagos University Teaching Hospital, Idi-Araba	37.5	5.6	<0.001	26.6	48.5
Mushin General Hospital	−15.6	5.3	<0.001	−25.9	−5.2
Onikan Health Care Centre	6.4	5.9	0.28	−5.2	17.9
Orile Agege General Hospital	5.1	5.3	0.33	−5.3	15.5
Randle General Hospital (Gbaja-Surulere MCC)	−14.2	5.8	0.01	−25.5	−2.8
Somolu General Hospital	−15.3	5.6	0.01	−26.4	−4.2
_cons	27.6	4.6	<0.001	18.5	36.6
**Distance (kilometres)**	**Coef.**	**Std. Err.**	***P* > |t|**	**[95% CI]**	
**Facility of care** ^a^					
Ajeromi Ifelodun General Hospital	−5.9	2.3	0.01	−10.5	−1.4
Alimosho General Hospital	2.9	2.3	0.19	−1.5	7.4
Amuwo-Odofin MCC	−3.1	2.6	0.24	−8.2	2.0
Apapa General Hospital	−6.0	2.4	0.01	−10.7	−1.4
Badagry General Hospital	6.7	2.5	0.01	1.9	11.6
Epe General Hospital	2.2	2.1	0.31	−2.0	6.3
Eti-Osa MCC	−2.7	2.3	0.23	−7.2	1.8
FMC Ebute-Metta	−2.5	2.5	0.31	−7.3	2.3
Gbagada General Hospital	−2.7	2.6	0.29	−7.8	2.3
Harvey Road Health Centre	−6.5	2.3	0.004	−11.0	−2.0
Ibeju-Lekki General Hospital	16.5	2.3	<0.001	11.9	21.0
Ifako-Ijaiye MCC	0.0	2.6	0.99	−5.0	5.0
Ijede Health Care Centre	−0.3	2.5	0.90	−5.3	4.7
Ikorodu MCC	−4.0	2.1	0.06	−8.2	0.2
Isolo MCC	−4.9	2.3	0.033	−9.3	−0.4
Lagos Island Maternity Hospital	8.2	2.1	<0.001	4.1	12.4
IMCH—Àyìnkę House	7.8	3.1	0.01	1.7	13.9
Lagos University Teaching Hospital, Idi-Araba	2.5	2.4	0.29	−2.2	7.3
Mushin General Hospital	−9.5	2.3	<0.001	−14.0	−5.0
Onikan Health Care Centre	−1.0	2.5	0.69	−6.0	4.0
Orile Agege General Hospital	−0.2	2.3	0.91	−4.8	4.3
Randle General Hospital (Gbaja-Surulere MCC)	−8.3	2.5	<0.001	−13.3	−3.4
Somolu General Hospital	−8.9	2.4	<0.001	−13.7	−4.1
_cons	13.4	2.0	<0.001	9.5	17.3

a Agbowa General Hospital was reference point.

There was a significant reduction in mean travel time and distance to health facilities for pregnant women who lived within the cluster surrounding the newly constructed Eti-Osa MCC before and after its commissioning [(−42 (95% CI −47–36) minutes)] and after [(−25 (95% CI −27–22) km)] the commissioning of the facility (*P *< 0.001).

## Discussion

In this study, we set out to estimate more realistic travel time estimates for pregnant women in need of CEmOC using Google Maps and then use these estimates to assess geographical accessibility and coverage of CEmOC in Lagos state, Nigeria. By pioneering the application of an already available, accessible and scalable GPS navigation application that offers traffic, road condition and travel updates (Google Maps) to estimate travel time at scale and across a specific sub-national setting, we have been able to demonstrate that such tools can be particularly useful from a supply-side perspective to understand true geographic accessibility and coverage of CEmOC in an LMIC setting.

We found that total travel time ranged from 2 to 240 minutes for women who travelled directly to their chosen CEmOC facility and 7 to 320 minutes for women who sought care there because of a referral. Our findings are wider than self-reported travel time estimates reported by pregnant women in a qualitative study conducted in Lagos, which suggested that they required between 5 and 240 minutes to reach CEmOC facilities (Banke-Thomas *et al.*, 2020). A wider range of between 10 minutes to 1 day has been reported in a systematic review of qualitative studies on maternal emergency transport in LMICs (Wilson *et al.*, 2013). When our time estimates were compared with the WHO and other widely used benchmarks (WHO, UNFPA, UNICEF *et al*., 2009; Chowdhury *et al.*, 2017; Niyitegeka *et al.*, 2017; Ouma *et al.*, 2018), we found that in Lagos, more than 80 and 96% of pregnant women with obstetric emergencies, irrespective of referral status, were able to reach facilities within the 1 and 2-hour thresholds, respectively. It also fulfils the minimum Lancet commission recommended target of 80% coverage (Meara *et al.*, 2015). Our estimate in Lagos state, the most urbanized part of Nigeria, is not vastly different from a modelled estimate that reported that more than 90% of women of childbearing age in Nigeria reside within 2-hour travel time of a public hospital that they can access if in a situation of emergency (Ouma *et al.*, 2018). However, such high coverage levels may not hold in the sparsely populated north of the country where CEmOC facilities are few and far between (Kabo *et al.*, 2019).

Our study also showed that regardless of the day of presentation (weekend or weekday), those travelling in the morning, afternoon or evening were more likely to travel longer than 60 minutes to reach a CEmOC facility compared to those that travel at night. This observation might be related to the significant ‘go-slow’ traffic that is a feature of Lagos during the morning and evening peak periods (Asiyanbola *et al.*, 2012). The traffic has been linked to poor road conditions, dense population, inadequate road network, poor traffic management and disorderly driving by many commuters in Lagos. In addition, our study also highlighted that referral significantly increased the odds of pregnant women needing more than 1 hour to reach CEmOC facilities, more so for those referred from other public CEmOC facilities (general hospitals and MCCs). While this might be intuitively right, as public CEmOC facilities would typically be further away from each other compared to PHCs and private hospitals (many of which feed into CEmOC facilities around them), such prolongation of travel time to health facilities might also be due to poor/inefficient referrals as has previously been reported in the literature (Cham *et al.*, 2005; Chi *et al.*, 2015; Banke-Thomas *et al.*, 2020). The additional insight from our study is that CEmOC-to-CEmOC referrals do occur, with 30-54% of referrals to teaching hospitals originating from general hospitals. Women have a positive opinion of public CEmOC facilities in Lagos due to its perceived conglomeration of highly skilled health personnel (Wright *et al.*, 2017). However, it might be the case that while technical capacity may be available, infrastructural capacity in terms of bed space and equipment (e.g. incubators to manage preterm babies) may be lacking in these general hospitals, explaining why some women seeking care there require onward referral.

Following geocoding, it appears many of the women in the central cities and towns to the east and west of the state mostly tended to travel to CEmOC facilities within and around their geographical area. However, we identified four cluster areas from which many women needed more than 60 minutes to reach CEmOC facilities. The newly commissioned Eti-Osa MCC (Bassey, 2019) was located in one of these clusters, and evidence from our before-and-after analysis shows that it had led to a significant reduction in travel time. From our previously published qualitative enquiry with women using this facility, many travelled to LIMH or Ibeju-Lekki General Hospital before its launch (Banke-Thomas *et al.*, 2020). In the other three clusters, poor road conditions, including flooding due to blocked drainages and incomplete road construction, have been a huge challenge to travel in the recent past (Adonai, 2020; Hanafi, 2020). In addition, a high number of referrals from these facilities based in the suburbs to the city may also be a massive contributor to the increased travel time experienced by women. In a previous study, the Lagos State Government stated that CEmOC facilities have been ‘strategically located across the state’. However, many women still report difficulty in timely access to facilities (Banke-Thomas *et al.*, 2017). Some researchers have suggested that these difficulties could be related to poorly located EmOC services (Niyitegeka *et al.*, 2017) or the insufficient number of facilities within a reasonable distance for travel (Mkoka *et al.*, 2014). While some researchers have identified catchment areas with models (Chowdhury *et al.*, 2017), our use of travel time estimates from Google Maps showed that these ‘left-behind’ catchments (clusters, as we have called them) could appear and disappear dynamically, varying within the time of the day and highly responsive to the construction of new facilities. Our approach certainly helps in realizing some of the ‘ambitions’ for defining accurate and representative service catchment areas for public services, as described in a recent commentary (Macharia *et al.*, 2021).

Some policy implications need to be considered based on our findings. First, pregnant women in the suburbs should remain encouraged to use CEmOC facilities close to them while ensuring that those facilities have the technical and infrastructural capacity to provide the full scale of CEmOC 24/7. In the four clusters identified as coverage gaps, there are varied explanations for the higher occurrence of prolonged travel in these areas, necessitating targeted responses to address the prolonged travel to access CEmOC facilities. For Clusters A (Alimosho/Ifako-Ijaiye) and D (north of Ikorodu), there are established CEmOC facilities within these areas already and it appears the challenge might be their relative inaccessibility. To address this gap, a mix of road expansion and repair as well as optimization of referral systems could be effective to minimize travel time. The construction of the new facility in Cluster B (Eti-Osa) during the data collection period is an excellent case study in the effectiveness of such a strategy in addressing travel delays. This should be continually monitored to ensure women continue to use the nearby facility. There is also a need to understand why women bypass nearby facilities. For Cluster C (Ijanikin/Morogbo), on-going large-scale road constructions should be concluded sooner rather than later, to minimize further travel delays. In addition, there is no CEmOC facility for about 30 km to the east and west of this cluster. The construction of a new CEmOC facility might be a priority intervention for the government to consider.

In terms of the other policy options across the state, expansion of ambulance services has been recommended as a means to improve referral (Tsegaye *et al.*, 2016), with researchers suggesting that its lack thereof hampers timely access to facilities (Geleto *et al.*, 2018). While this might be the case in certain instances, the traffic contributes to delays for Lagos ambulances in 60% of cases (Venkatraman *et al.*, 2020). In addition, other drivers do not tend to give way to ambulances (Adewole *et al.*, 2012). Addressing these two limitations through traffic enforcement and education of drivers should be done alongside any investments in purchase of ambulances. Indeed, there is the option to expand the capacity of BEmOC facilities, such as primary health care centres across all clusters that have referral-related delays and are located farther away from the tertiary facilities. However, BEmOC facilities do not provide the full scale of care that women may require in emergency situations, and many of such facilities have low delivery volume, which makes skill retention of the health workers unlikely (Adegoke *et al.*, 2012). This means that a higher percentage of pregnant women presenting in these facilities need to be referred, thereby prolonging total travel time and the time before they receive lifesaving EmOC. Ultimately, such delays increase the risk for poor pregnancy outcomes for mothers and their newborns (Bossyns *et al.*, 2006; Elmusharaf *et al.*, 2017). Authors of a study that modelled the geographic feasibility of service delivery redesign in six LMICs suggested focusing on building the capacity of CEmOC facilities instead of BEmOC facilities, highlighting that such a policy ‘would not unduly affect geographical access’. Their model estimated that such a policy would reduce the percentage of those who can access facilities within 2 hours by at most 10%. At the 1-hour threshold, 4–20% of women would exceed 1-hour travel time access to CEmOC facilities, with a greater effect on women who reside in remote areas (Gage *et al.*, 2019). As evidence suggests that every 5-minute increase in travel time even to the nearest EmOC facility is associated with a 30% decrease in the coverage of the percentage of births occurring in health facilities, favouring home-based care (Panciera *et al.*, 2016), such policy recommendation needs to be carefully considered.

At a global level, there is a need to review the guidance on the 2-hour travel time benchmark and 80% minimum target coverage (Meara *et al.*, 2015). While such targets might have been set with the objective of realism (WHO, UNFPA, UNICEF *et al*., 2009), it does not fit into the current global goals to ‘leave no one behind’ and to achieve universal health coverage (UN, 2020; WHO, 2020). In any case, it begs the question—‘what about the remaining 20%’? Should they not be able to access critical services in good time? The other critical challenge is that travelling to reach a facility includes deciding to go to the facility, finding appropriate means of transport and getting ready to go (Banke-Thomas *et al.*, 2020). All these micro-phases are not accounted for in these recommended benchmarks. In addition, it is known that the most rapidly fatal pregnancy complication, haemorrhage, can lead to maternal death within 2 hours of starting and for some women even in minutes (Khan and El-Rafaey, 2006; UNFPA, 2012). In one model-based study, the authors showed that 16 sub-Saharan countries are already achieving the 80% 2-hour travel time target (Ouma *et al.*, 2018), so certainly this threshold is attainable and yet travel to facilities remain the significant contributor to maternal death. It might be time to set the bar higher.

For practice implications, while we have been able to push the frontiers of the field and shown what is possible, health information management systems still have to be set up to collect full and accurate data on the travel of all pregnant women as part of routine history-taking. We depended heavily on the high levels of completeness and accuracy of data on the origin and referral points in patient records. Where there were gaps or confusing entries, medical doctors who served as data collectors in our study were able to clarify from the actual care providers to complete data. However, in some instances, this was not possible. At the barest minimum, addresses, names of referral points and other stopovers made while travelling to the facility need to be reported for every pregnant woman.

Overall, our study clearly shows that when closer-to-reality travel time estimates are available and aggregated, they will generate highly relevant insights that identify specific areas of geographical inequity. This lines up with long-held expectations on the capacity of GIS to identify the optimum location of new/upgraded facilities (Admasu *et al.*, 2011; Banke-Thomas *et al.*, 2016). The additional insight that our study provides also addresses some of the key gaps that policymakers and researchers have deemed necessary to reach ‘utopia’ for geospatial analysis for reproductive, maternal, newborn, child and adolescent health (Matthews *et al.*, 2019). While we have used Google Maps in our study, other similar platforms are increasing in popularity now. For example, Waze (https://waze.com) developed by Waze Mobile, which is similar in functionality to Google Maps. Here WeGo (https://wego.here.com/) developed by HERE Global B.V. allows real-time traffic, public transport, pedestrian and bicycle navigation route functions. These proprietary platforms usually come at a cost, especially if a significant number of queries are being requested for analyses. Such costs could be prohibitive for LMIC researchers (Banke-Thomas *et al.*, 2019). However, there is also an open-source, World Bank supported application called OpenTraffic (http://opentraffic.io/) that also captures real-time traffic. On the flip side, as a demand-side strategy to assess travel time, some researchers see an opportunity to mine data being gathered from smartphones equipped with GPS functioning and collated by big-tech companies (Weiss *et al.*, 2020). While this is certainly the future, the feasibility of this demand-side strategy for LMICs is probably questionable for now, with smartphone penetration still between 13 and 51% in many sub-Saharan African countries (Silver and Johnson, 2018).

Our findings need to be interpreted while bearing in mind some key limitations. First, while we have leveraged actual patient data to map the travel paths of the pregnant women, we have not captured the travel time based on their actual ‘experienced’ travel time. However, Google Maps has been shown to be 85% (IQR = 69–98%) accurate in reflecting actual travel time (Banke-Thomas *et al.*, 2021). For the travel time estimates that we had, it was impossible to estimate travel time for journeys that included waterways, as Google Maps does not have this capacity. There were eight such cases in our dataset. However, there may be more who were classed as ‘untraceable’ because there was no address in their records or we could not find their stated address. Some of these women may be living in very remote settlements and slums including riverine ones like Makoko, where higher mortalities have been reported (Anastasi *et al.*, 2017). Tracing addresses in such settlements is an impossible task, although about 40% of those who live in these settlements tend to use private health facilities close to their communities (Anastasi *et al.*, 2017). In addition, although we had data on the months of presentation and could have aggregated to assess seasonal patterns that may affect travel, we were also not able to do this as Google Maps does not have the capacity to show monthly variation in travel time estimates. We have also assumed that women commuted by a motorized vehicle to the facility and not estimated travel time for women who might have walked to the health facility—a means of travel taken by 30% of women in Lagos, as per the 2018 NDHS (National Population Commission, ICF International, 2019). In addition, we have not included the additional time that it could have taken to prepare means of travel to the facility. This suggests that our time estimates may be shorter than the reality experienced by some women. Furthermore, our study was based on women who made it to a public CEmOC facility. There would have been those who went directly to a private hospital or died before arrival at a public hospital. Such cases are completely missing from our data. Finally, some facilities were not working to capacity when we collected the data due to repair works. However, we do not believe these would have significantly altered our findings.

## Conclusion

For pregnant women in emergency situations, reaching health facilities with the full repertoire of resources to provide the care they need is truly a case of every minute matters. Factors that are for the most part out of the control of pregnant women including the period of the day they travel, being referred (especially from another public CEmOC facility), and trying to reach the ‘big’ tertiary hospitals prolonged travel time. Clearly, more needs to be done in supporting pregnant women to reach care in emergencies. Presently, most of our understanding of women’s travel times to health facilities in many LMICs has thus far been based on conjectures far away from reality. With innovation clearly needed in capturing these data to promote equity in EmOC service access, our study shows that ubiquitous GPS navigation applications such as Google Maps, if deployed on a large scale can provide the critical, context-specific and closer-to-reality evidence that will allow policymakers to be more effective. Analyses based on such platforms will be more engaging for policymakers and if combined with supportive economic costing of policy options can significantly improve their decision-making capacity towards achieving an equitable distribution of health facilities not just for EmOC but for all health emergency services. However, actions taken to address any gaps identified need to be contextual and responsive to ‘hotspots’. Future research should look at the interaction between travel time, obstetric complication and pregnancy outcomes.

## Supplementary Material

czab099_Supp

## Data Availability

For the privacy of individuals that participated in the study, data underlying this article cannot be shared publicly.
