# Supplementary material for: An assessment of geographical access and factors influencing travel time to emergency obstetric care in the urban state of Lagos, Nigeria
Source: Health Policy Plan. 2021 Aug 23;36(9):1384–96. doi: 10.1093/heapol/czab099 (PMC8505861; doi:10.1093/heapol/czab099)
Supplement: czab099_Supp [file czab099_supp.zip › Supplementary data.pdf]

Supplementary Data: Additional information on Lagos CEmOC facilities

S/N	Facility name	Latitude (x)	Longitude (y)	Facility type	Year of establishment	Number of deliveries in 2018
1	Agbowa General Hospital	6.645643	3.712914	General Hospital	2003	157
2	Ajeromi Ifelodun General Hospital	6.455283	3.33362	Maternal and Childcare Centre	1983	825
3	Alimosho General Hospital	6.561196	3.250662	Maternal and Childcare Centre	2006	2,484
4	Amuwo-Odofin MCC	6.461779	3.301963	Maternal and Childcare Centre	2014	2,484
5	Apapa General Hospital	6.441943	3.368962	General Hospital	1964	120
6	Badagry General Hospital	6.413752	2.900374	General Hospital	1957	973
7	Epe General Hospital	6.586945	3.971665	General Hospital	1954	883
8	Eti-Osa MCC*	6.465421	3.585173	Maternal and Childcare Centre	2019	-
9	FMC Ebute-Metta	6.484271	3.380194	Tertiary Hospital	1964	1,820
10	Gbagada General Hospital	6.551919	3.387304	General Hospital	1983	1,729
11	Harvey Road Health Centre	6.50935	3.372629	General Hospital	1962	717
12	Ibeju-Lekki General Hospital	6.439312	3.934335	General Hospital	2006	982
13	Ifako-Ijaiye MCC	6.680364	3.291382	Maternal and Childcare Centre	2006	2,916
14	Ijede Health Care Centre	6.564649	3.596596	General Hospital	1983	869
15	Ikorodu MCC	6.608265	3.499394	Maternal and Childcare Centre	1983	2,820
16	Institute of Maternal and Child Health - Àyìnkę House*	6.590868	3.34092	Tertiary Hospital	1955	-
17	Isolo MCC	6.527787	3.318979	Maternal and Childcare Centre	1975	2,147
18	Lagos Island Maternity Hospital	6.448543	3.397417	Tertiary Hospital	1960	3,681
19	Lagos University Teaching Hospital	6.517803	3.353786	Tertiary Hospital	1962	2,011
20	Mushin General Hospital	6.531566	3.348531	General Hospital	1954	524
21	Onikan Health Care Centre	6.444656	3.405436	General Hospital	1971	544
22	Orile Agege General Hospital	6.635353	3.303379	General Hospital	1982	1,677
23	Randle General Hospital (Gbaja-Surulere MCC)	6.50433	3.359574	Maternal and Childcare Centre	1960	2,056
24	Somolu General Hospital	6.536461	3.372251	General Hospital	2006	619

*Facilities not running in 2018. As such no details available on number of deliveries.
