# Supplementary figures and images for: An assessment of geographical access and factors influencing travel time to emergency obstetric care in the urban state of Lagos, Nigeria
Source: Health Policy Plan. 2021 Aug 23;36(9):1384–96. doi: 10.1093/heapol/czab099 (PMC8505861; doi:10.1093/heapol/czab099)

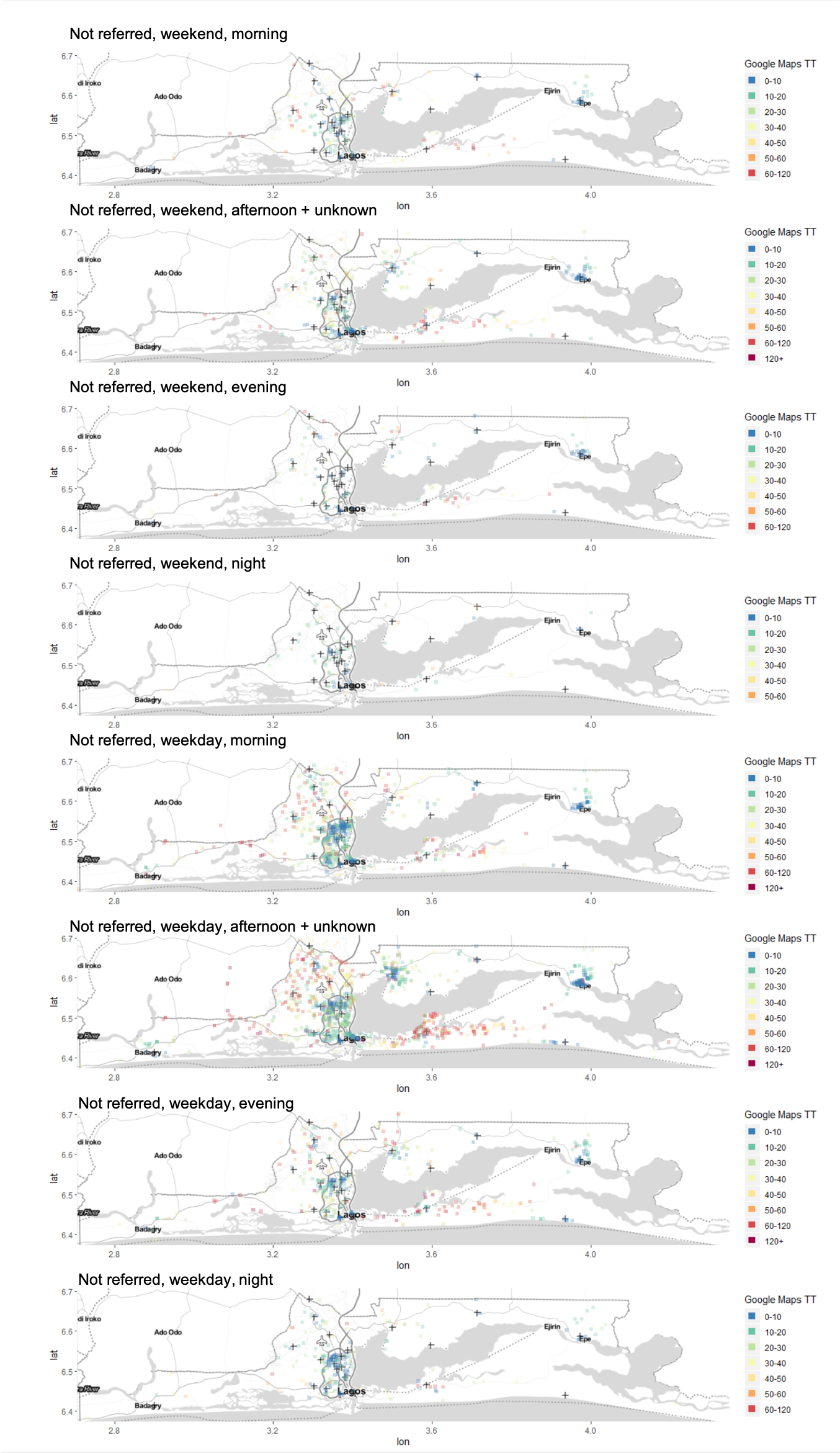

Supplement: czab099_Supp [file czab099_supp.zip › Suppl_figure_1.tiff]

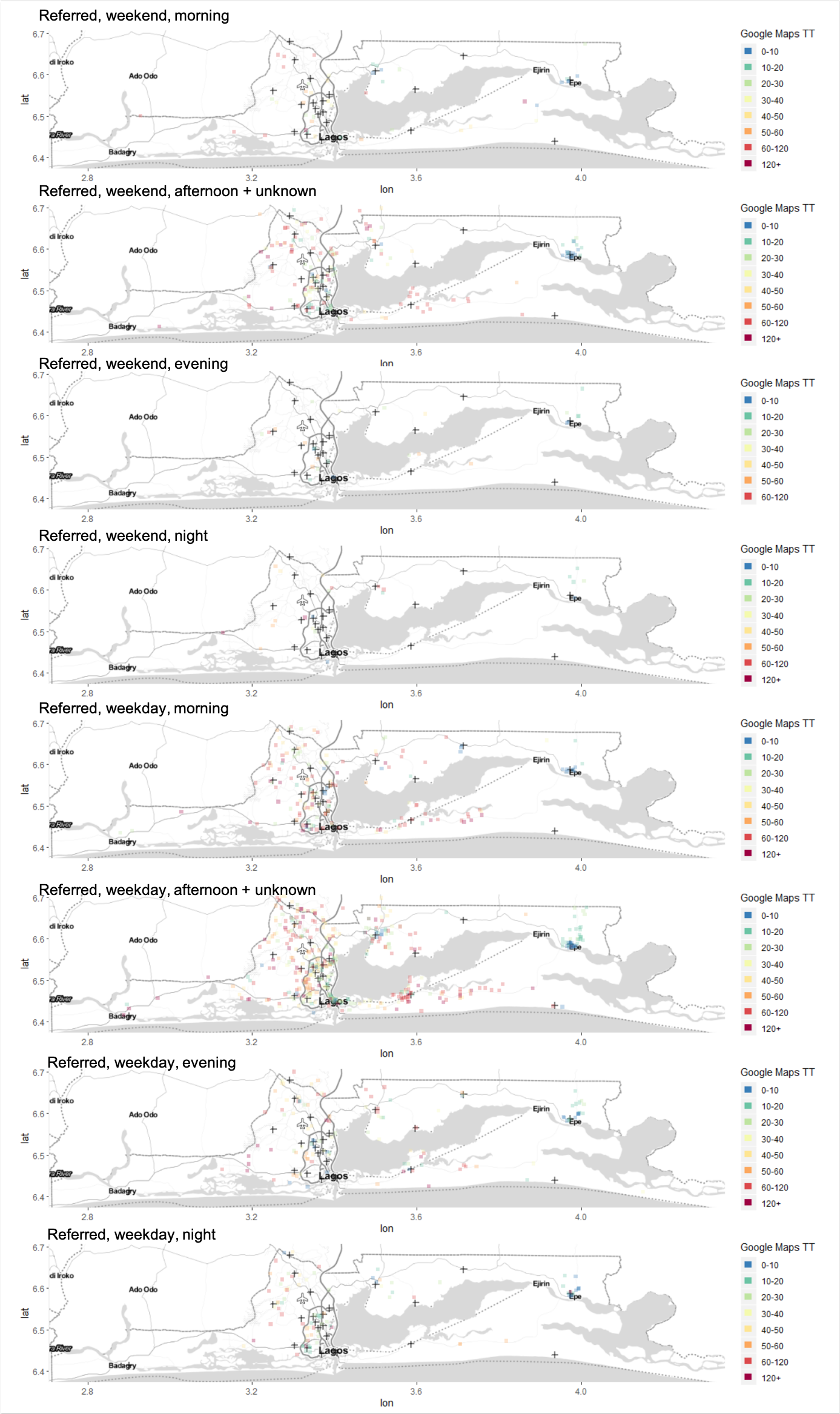

Supplement: czab099_Supp [file czab099_supp.zip › Suppl_figure_2.tiff]
